# The effect of environmental factors in childcare facilities and individual lifestyle on obesity among Japanese preschool children; a multivariate multilevel analysis

**DOI:** 10.1097/MD.0000000000017490

**Published:** 2019-10-11

**Authors:** Motohide Goto, Yukiyo Yamamoto, Reiko Saito, Yoshihisa Fujino, Susumu Ueno, Koichi Kusuhara

**Affiliations:** a Department of Pediatrics, School of Medicine; b Department of Occupational Toxicology, Institute of Industrial Ecological Sciences; c Director of Medical Education, School of Medicine; d Department of Environmental Epidemiology, Institute of Industrial Ecological Sciences, University of Occupational and Environmental Health, Yahatanishi-ku, Kitakyushu, Japan.

**Keywords:** childcare facilities, childhood obesity, multilevel analysis, receiving snacks in facilities, self-administered questionnaires

## Abstract

Lifestyle in preschool children is associated with the onset of childhood obesity. However, the effect of environmental factors in childcare facilities on lifestyle and obesity in preschool children is unknown. The aim of this study was to determine the effect of environmental factors in childcare facilities on the association between obesity and individual lifestyle in preschool children.

Subjects included 2902 infants, aged 4 to 6 years old in Kitakyushu City, Japan. A stratified multilevel analysis was conducted with 2 strata: factors related to individual lifestyle and maternal factors as the individual level and factors related to the childcare facility as the environmental level. Two-level multilevel regression analysis was conducted with the presence or absence of obesity.

The proportion of infants with obesity was 4.2%. The childhood obesity was significantly associated with the mastication, nutritional methods during infancy, absence of breakfast, presence of skipping meals due to overeating of snacks, usual play activity, screen time on weekdays, maternal body mass index, and maternal weight increase during pregnancy at the individual level. On the other hand, childhood obesity had a significantly negative association with the receiving snacks in facilities by using multilevel analysis.

The present study revealed that establishing and maintaining environmental factors in childcare facilities may play important roles in the prevention of obesity from early childhood.

## 1 Introduction

Childhood obesity is prevalent in many countries and associated with poor physical and psychosocial health across the life course.^[1–3]^ One of the most significant problems with childhood obesity is that it frequently leads to adulthood obesity and the associated risks for various diseases in adulthood such as diabetes, lipid metabolism disorder, or arteriosclerosis.^[4–8]^ Further on, these conditions may lead to the development of ischemic heart disease or cerebral infarction in adulthood.^[3]^ Therefore, childhood obesity that leads to adulthood obesity should be prevented for lifetime health.

Lifestyle and maternal factors in early childhood have been associated with the development of childhood obesity.^[9]^ Previous studies document that maternal diabetes, infantile obesity, infantile low body activity, short sleeping period, and soft drink consumption in early childhood are associated with the onset of childhood obesity.^[9–12]^ Therefore, identification of individual lifestyle or maternal factors, and prompt intervention would be effective for the prevention of childhood obesity.

Alongside individual lifestyle factors, behavioral and environmental factors are also implicated in childhood obesity. Among environmental factors, several studies site household income, levels of education or residential location are significantly associated with the onset of childhood obesity.^[5,13–16]^ Environmental factors in childcare facilities, where preschool children spend most of daytime, are expected to have significant influence on the individual factors.^[17]^ However, there are no previous studies about the association between childhood obesity and the characteristics of care provisions such as the presence of gardens, extracurricular activities, or meal contents in preschool. Identification of environmental factors that affect the onset of childhood obesity may help prevent the onset of childhood obesity. Standard statistical procedures using multivariate regression model demands individual or environmental independence. Combination tests of individual factors and environmental factors are more useful in investigating factors contributing to the onset of childhood obesity. The aim of this study is to investigate the effects of environmental factors in childcare facilities on the association between childhood obesity and lifestyle.

## 2 Methods

### 2.1 Data

This study is a cross-sectional study. This study was examined the kindergartens and nursery schools in Kitakyushu City, and questioned about individual factors in December 2012 and asked about environmental factors in October 2014. Of the subjects (3170) who completed a questionnaire, 2902 children aged 4 to 6 years (5.16 ± 0.68) had complete data. The questionnaire response rate was 95.7%. They were recruited from 56 facilities (kindergartens and nursery schools) in Kitakyushu City. The participants and facilities were selected by the nonrandom method. The child with obesity was defined if his/her percentage overweight (POW) exceeded 20% based on the age- and sex-specific standard body weight for the height. POW was calculated as 100 × (the measured weight – normal weight)/normal weight (%). Although POW is unique to Japan, it is not influenced by height, therefore, it is a highly useful index for longitudinal studies and has been widely used in school or preschool health checkups to evaluate children's weight periodically.^[18,19]^ Normal weight was based on age-and sex-specific standard body weights for height were obtained from the Ministry of Education, Culture, Sports, Science, and Technology. The Japanese criteria of childhood obesity and mild obesity is classified as mild obesity when POW is 20% or more and less than 30%, moderate obesity is POW 30% or more and less than 50%, and severe obesity is classified as POW 50% or more.^[18,19]^ Self-administered questionnaires were distributed to the kindergartens and nursery schools, and the questionnaires were completed by the parents anonymously and collected by the staff in each facility. Subsequently, a self-administered questionnaire regarding environmental factors in the facilities was distributed to each facility, and the questionnaires were completed by the staff in each facility.

### 2.2 Individual-level variables

The questionnaire consisted of questions regarding characteristics of the child (body height, weight, sex, birth height, and weight), dietary habits (presence or absence of skipping meals, meal times, presence or absence of sweet bun as a staple food, vegetable intake status, regularity of snack times, presence or absence of skipping meals due to overeating of snacks, proper chewing of food, feeding method in infancy), exercise activity (daily playing activity, time spent watching TV), and maternal factors (maternal body height and weight during pregnancy, maternal weight gain during pregnancy, maternal employment status). The height and weight of the children were measured and recorded by staff at each facility. The information on maternal factors such as maternal body height and weight during pregnancy, maternal weight gain during pregnancy and maternal employment status were collected by the answer of the questionnaire.

### 2.3 Environmental-level variables

The questionnaire addressed the indoor and/or outdoor play space, time for physical activity, limited extracurricular activities, presence or absence of nap time, receiving a second serving at lunch time, receiving snacks in facilities, presence or absence of anthropometric measurements.

### 2.4 Statistical analyses

Data were presented as the mean ± standard deviation. Binary logistic regression was done to investigate the association between explanatory variables and outcome variable (childhood obesity). Explanatory variables such as individual factors and environmental factors were included in the binary regression. Of these, explanatory variables which had crude odds ratios at 95% confidence interval (CI) and *P*-value of less than .05 on the bivariate analysis were included in the final multiple logistic regression model to control for confounders. Multicollinearity of the independent variables was checked by standard error, and variables with standard error of greater than 2 were excluded from the multivariate analysis. Multilevel analysis was used to investigate the possible factors in response to treatment in childcare facilities. For data statistical analysis, a stratified analysis was performed to analyze the relationship between individual factors related to lifestyle (individual level) and environmental factors in childcare facilities (environmental level). A hierarchical linear model analysis was performed with the presence or absence of obesity as dependent variables, and individual level and environmental level as independent variables. The statistical analyses were performed using STATA version 13 (StataCorp, College Station, TX). This study was approved by the Ethics Committee of Medical Research, University of Occupational and Environmental Health, Japan. Written informed consent was obtained from the study participants, including consent to participate and to publish the findings.

## 3 Results

The characteristics of subjects are presented in Table 1. Of them, 122 (4.2%) children were diagnosed with obesity. Among 122 children with obesity, 71 (2.4%) had mild obesity, 42 (1.4%) for moderate obesity and 9 children with obesity (0.3%) for severe obesity. The characteristics of environmental factors in facilities are presented in Table 2.

**Table 1:**
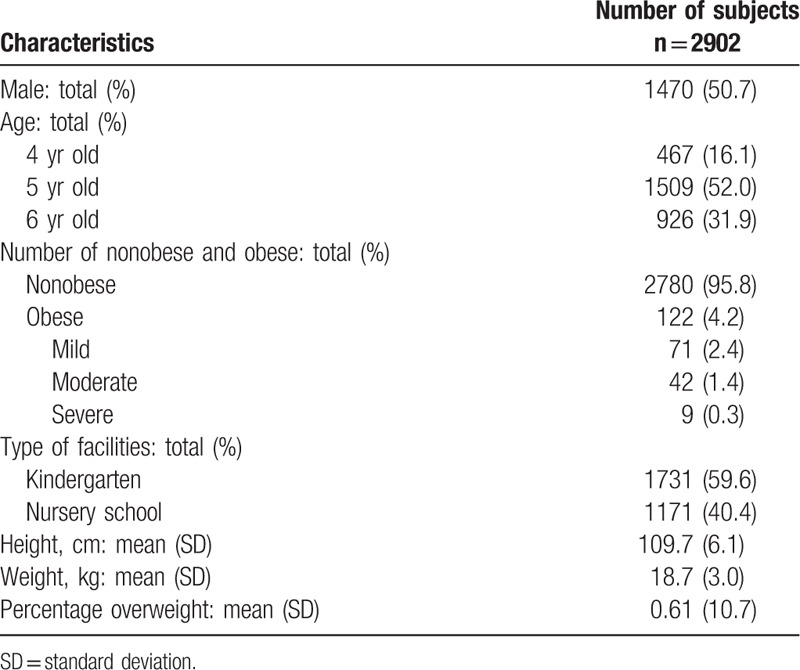
Characteristics of the subjects.

**Table 2:**
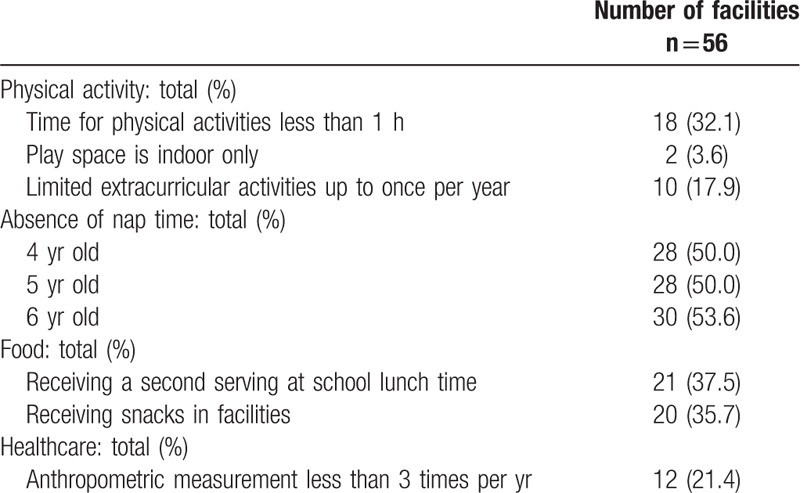
Characteristics of environmental factors in facilities.

The result of individual-level by using multivariate multilevel analysis are presented in Table 3. Among individuals, the prevalence of obesity was significantly associated with mastication habits (poor chewing habits), feeding method in infancy (bottle-fed formula), absence of breakfast, presence of skipping meals due to overeating of snacks, daily playing activities (only watching TV or playing TV games), time spent watching TV more than 2 hours per day on weekdays, maternal body mass index (BMI) more than 25, and maternal weight gain during pregnancy more than 20 kg.

**Table 3:**
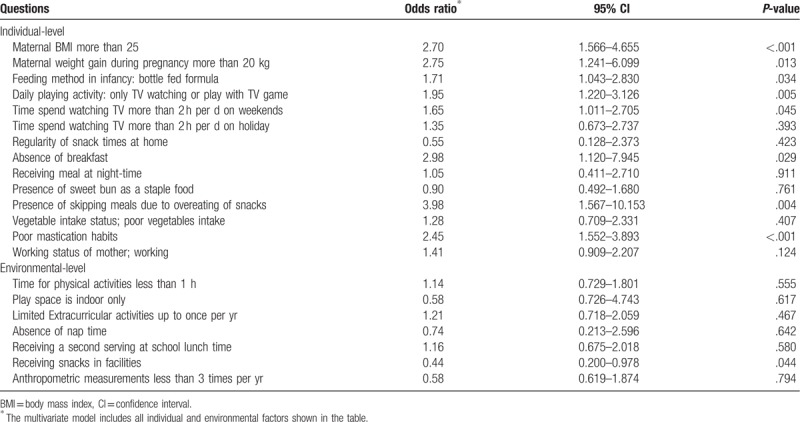
Odds ratios of individual factors and environmental factors for childhood obesity.

The results of environmental level data by using multivariate multilevel analysis are presented in Table 3. The multilevel analysis found that only those children who receiving snacks in facilities were significantly associated with the prevalence of obesity (odds ratio = 0.44, 95% CI: 0.200–0.978, *P* = .044).

## 4 Discussion

We revealed 2 notable points in this study. First, we demonstrated a more accurate model-based upon multilevel analysis for the verification of both the influence of individual lifestyle as well as environmental factors on childhood obesity. Thereby, we have identified that adjusting environmental factors at childcare facilities may be useful for the prevention of childhood obesity. Second, the result of our study showed that childhood obesity was significantly associated with receiving snacks in childcare facilities. We suggest that taking regular and pre-determined sizes of snacks may reduce the rates of childhood obesity.

We examined the relationship between childhood obesity and individual lifestyle, such as dietary habits and physical activity. In dietary habits, some reports previously demonstrated a significant association between mastication and childhood obesity and suggested that the mastication movement was acquired through the process of advancing baby food, and then gradually developed further with the mastication habits in early childhood.^[20,21]^ Monzani et al reported that skipping breakfast was significantly associated with childhood overweight and obesity, and they also revealed that skipping meal was associated with a worse lipid profile, blood pressure levels, insulin-resistance, and metabolic syndrome.^[22]^ Thus, our study which using stratified multilevel analysis also revealed that there is a significant association between eating habits and childhood obesity. Our study also showed significant association between physical activity and childhood obesity. Ochiai et al revealed that the incidence of obesity increased significantly in the school children with a TV viewing time of 4 hours or more and a video game of 2 hours or more.^[23]^ Due to changes in lifestyle even in early childhood, time for outdoor play has decreased, and play by television and video game is increasing. These changes influenced not only decreasing the amount of physical activity, but also increasing the snacking due to the influence of fast food and TV advertisement of food.^[24]^ These findings indicate that individual lifestyle acquired in early childhood may have a great influence on the onset of childhood obesity and appropriate lifestyle should be started even from early childhood.

The present study showed that the efforts at childcare facilities were significantly related with the lower rate of obesity in preschool children. By using multilevel analysis with individual and environmental factor data, we revealed that childhood obesity was significantly associated with the receiving of snacks in facilities. Regarding with the association between childhood obesity and environmental factors in school, several studies have reported that the household income, levels of education or residential area were significantly associated with the onset of childhood obesity.^[5,13–16]^ There have been no studies about the association between environmental factors such as intervention at childcare facilities and childhood obesity. The present study showed that critical examination of the environmental factors in childcare facilities, where children spend most of daytime, could be effective in the prevention of childhood obesity even from early childhood. These results suggest that educational activities should be conducted through kindergartens and nursery schools, and that active intervention and guidance is necessary.

The association between certain snacking behaviors and increased rates for the onset of childhood obesity is well documented. There are also studies reporting that intake of a regular and determined amount of snacks has a positive effect, suppressing the rates of the onset of childhood obesity.^[25–27]^ The National Health and Nutrition Examination Survey (NHANES) examined the association between snack intake factors (frequency of snack intake and the percentage of energy taken from snacks) and obesity factors (degree of obesity and abdominal circumference) in 5811 children aged 12 to 18 years from 1999 to 2014, and reported that mean body weight, abdominal circumference, and frequency of obesity were significantly higher in the group without snacks.^[25]^ Furthermore, the NHANES also investigated the association of snack intake status with the degree of obesity and abdominal circumference in 7049 children aged 2 to 13 years and reported that the rates of overweight (85–95 percentile) and children with obesity (≥95 percentile) were higher in the group without snacks than in the group with snacks.^[26]^ Previous studies reported that snacks containing fruit, whole-grain, and dietary fiber reduced the risk of obesity.^[27]^ To prevent obesity, the American Academy of Pediatrics recommends structured “healthy and nutritious” snacking, as opposed to ongoing grazing, suggesting that elementary-aged children have 1 to 2 snacks daily and toddlers up to 3 snacks.^[28]^ Ingestion of regular and controlled snacks may be useful for suppression of childhood obesity. In the present study, the contents of snacks were not examined. Further detailed studies would be needed for the investigation for the effect of quality of snacks.

The present study showed that the environmental factors at childcare facilities had significant influence on the effect of individual lifestyles for childhood obesity. Most importantly, multilevel analysis was successfully applied in the analysis of childhood obesity. By means of the relevant data analysis, the risk factors that affected the onset of childhood obesity could successfully be investigated. By comparing the individual lifestyle with the environmental factor of each facility, it is possible to specifically evaluate the influence of items related to obesity onset.

Maternal factors also have a great influence on the onset of childhood obesity. Indeed, we revealed the relationship between childhood obesity and maternal factors such as working situation or physical condition during pregnancy by using logistic regression analysis. A previous report revealed that children of mothers with obesity tend to be 2.6 times more obese than children with nonobese mothers.^[29]^ Weight gain during pregnancy has been reported to affect maternal insulin resistance and hormonal secretion in the womb and obesity in postnatal infants.^[20]^ Therefore, it is important to provide guidance about appropriate weights during pregnancy for the prevention of childhood obesity or maternal lifestyles for the acquisition of appropriate lifestyle since early childhood.

We have some potential limitations of the present study. First, we could not include all nursery schools in the study. While we examined almost all of the kindergartens and licensed nursery schools in Kitakyushu City were covered, unlicensed nurseries did not participate. It is possible that some important factors are being overlooked by leaving out these facilities. Moreover, it may be over adjustment to use all factors for logistic regression analysis because the number of obesity is small. We need to consider the opportunity to expand the examination in the future to 100% of the childcare facilities throughout Kitakyushu City. Second, we could not collect well-known environmental variables such as parental weight or height, household income, levels of education or residential location which are considered to be related with the onset of childhood obesity. We could collect the information on maternal factors such as maternal body height and weight during pregnancy, maternal weight gain during pregnancy by the answer of the questionnaire in this study, but this may introduce bias like recall bias which may affect the validity of the results. Moreover, this study is the cross-sectional study, and further longitudinal investigation are needed. Lastly, this survey has not been able to investigate the actual content and amount of snacks. It is inferred that kindergartens and nursery schools offer snacks of quantity and quality based on the “guidelines for providing meals at child welfare facilities” of the Ministry of Health, Labor, and Welfare, but since the actual content has not been investigated. We were able to consider only a limited set of individual and environmental factors in this study. There is a need to add more items and re-examine the relevance on childhood obesity.

## 5 Conclusion

This study revealed that establishing and maintaining environmental factors in childcare facilities had played important roles for prevention of obesity from early childhood. Taking a determined amount of snacks on a regular time would lead to the acquisition of well-regulated lifestyle.

## Acknowledgments

The authors thank Kyoko Watanabe, Kazuko Umeki, and Yoko Fukuda for collecting data of the manuscript. The authors thank David Askew, PhD, for his critical reading and language editing of the manuscript.

## Author contributions

**Conceptualization:** Motohide Goto, Yukiyo Yamamoto.

**Data curation:** Motohide Goto, Yukiyo Yamamoto, Reiko Saito.

**Formal analysis:** Motohide Goto, Yoshihisa Fujino.

**Investigation:** Motohide Goto.

**Methodology:** Motohide Goto.

**Project administration:** Motohide Goto, Yukiyo Yamamoto.

**Resources:** Motohide Goto.

**Software:** Motohide Goto, Yoshihisa Fujino.

**Supervision:** Yukiyo Yamamoto, Yoshihisa Fujino, Susumu Ueno, Koichi Kusuhara.

**Validation:** Motohide Goto, Reiko Saito.

**Visualization:** Motohide Goto.

**Writing – original draft:** Motohide Goto.

**Writing – review and editing:** Motohide Goto, Yukiyo Yamamoto, Yoshihisa Fujino, Susumu Ueno, Koichi Kusuhara.

Motohide Goto orcid: 0000-0002-0674-0152.
